# Design and Mixing Analysis of a Passive Micromixer with Circulation Promoters

**DOI:** 10.3390/mi15070831

**Published:** 2024-06-27

**Authors:** Makhsuda Juraeva, Dong-Jin Kang

**Affiliations:** School of Mechanical Engineering, Yeungnam University, 280 Daehak-ro, Gyoungsan 38541, Republic of Korea

**Keywords:** circulation promoter, concave wall, convex wall, degree of mixing (DOM), submerged structure, mixing mechanism

## Abstract

A novel passive micromixer equipped with circulation promoters is proposed, and its mixing performance is simulated over a broad range of Reynolds numbers (0.1≤Re≤100). To evaluate the effectiveness of the circulation promoters, three different configurations are analyzed in terms of the degree of mixing (DOM) at the outlet and the associated pressure drop. Compared to other typical passive micromixers, the circulation promoter is shown to significantly enhance mixing performance. Among the three configurations of circulation promoters, Case 3 demonstrates the best performance, with a DOM exceeding 0.96 across the entire range of Reynolds numbers. At Re = 1, the DOM of Case 3 is 3.7 times larger than that of a modified Tesla micromixer, while maintaining a comparable pressure drop. The mixing enhancement of the present micromixer is particularly significant in the low and intermediate ranges of Reynolds numbers (Re<40). In the low range of Reynolds numbers (Re≤1), the mixing enhancement is primarily due to circulation promoters directing fluid flow from a concave wall to the opposite convex wall. In the intermediate range of Reynolds numbers (2≤Re<40), the mixing enhancement results from fluid flowing from one concave wall to another concave wall on the opposite side.

## 1. Introduction

Micromixers play a vital role in numerous fields such as chemical analysis, biomedical diagnostics, drug development, food safety control, and animal epidemic prevention, as they facilitate the homogenization of sample reagents on a microscale [1,2,3,4]. These devices are essential for reducing reagent consumption, accelerating mixing process, and achieving compact sizes [3,4]. Efficient and swift mixing at the microscale is therefore critical to meeting these goals.

Despite its significance, microfluidic mixing often presents challenges in achieving rapid and efficient evolution due to the limitations imposed by the diminutive scale [1,2]. Although various technologies have been proposed to overcome these challenges, microfluidic mixing remains an active area of research [1,2,3,4]. Consequently, the advancement of microfluidic industry heavily relies on the development of efficient micromixers. 

The microscale dimensions of micromixers lead to an increased surface-to-volume ratio, resulting in slow fluid velocities and a heavy reliance on molecular diffusion for mixing. Therefore, there is a growing demand for micromixer designs that can introduce more vigorous flow conditions to enhance the overall performance of microfluidic systems. 

Various micromixer technologies have been proposed to enhance mixing in microfluidic systems, broadly categorized as active or passive. Active micromixers employ movable elements or external energy sources to perturb fluid flow and induce chaotic fluid flow. The external energy sources include magnetic fields [5], acoustic fields [6], electric current [7], pressure pulsation [8,9], or thermal energy [10,11]. Although offering precise control over fluid behavior, active micromixers face drawbacks such as complex driving structure, high energy consumption, and expensive fabrication. Additionally they are more prone to failure during operation than passive micromixers. These hurdles restrict the practical application of active micromixers in microfluidic systems, particularly in cost-effective and portable setups [12].

On the other hand, passive micromixers utilize geometric structures to enhance molecular diffusion and/or chaotic advection for efficient mixing. Consequently, they do not have movable elements and external energy sources apart from the pressure drop needed to drive the flow. Because of this advantage of simple fabrication and cost-effectiveness, passive micromixers are widely employed across a diverse spectrum of microfluidic systems. Despite various passive micromixers having been proposed [13], researchers continue to develop new types of passive micromixers. Examples include a block in the junction [14], various baffles and grooves [15,16,17], reverse staggered herringbones [18], twisting of channel walls [19], modified Tesla structure [20,21,22], cross channel configuration of split-and-recombine (SAR) [23,24,25], convergent-divergent micromixers [26], lateral structures [27,28], and submerged planar structures [29,30]. However, it is noteworthy that most passive micromixers have demonstrated mixing improvement within a limited range of Reynolds numbers. 

In various biological and chemical applications of microfluidic systems, the demand for mixing time on the order of milliseconds highlights the necessity for micromixers that can effectively operate across a broad range of Reynolds numbers (Re < 100) [31,32,33,34,35]. Within this Reynolds number range, micromixing is governed by two distinct mechanisms: molecular diffusion and chaotic convection. Therefore, three different regimes emerge based on the dominant mechanism: diffusion domination, transition, and convection domination. Among these three regimes, mixing in the transition regime experiences the least efficient performance, with corresponding Reynolds number typically ranging from 0.5 to 20. In this regime, both of molecular diffusion and chaotic convection play equally significant roles. Overcoming this limitation requires a novel design to enhance mixing performance by addressing both chaotic convection and molecular diffusion. 

To enhance mixing in the transition regime, some researchers have explored intricate three-dimensional (3D) structures. For instance, Xia et al. [36] introduced a two-layer crossing channel and obtained a noticeable mixing improvement at Re = 0.2. Yang et al. [21] proposed three-dimensional Tesla structures for bio-applications. A mixing enhancement was achieved by recurring separation and amalgamation of fluid streams, producing transverse dispersion in the crosswise plane within Tesla unit pairs. The improvement in mixing efficiency is up to 0.94 over a wide range of Reynolds numbers (0.1≤Re≤100). Hossain et al. [37] designed a passive micromixer based on three-dimensional serpentine SAR microchannel, resulting in a mixing index of 0.884 for Re = 30. However, 3D micromixers is expensive and difficult to fabricate compared to planar micromixers. Consequently, many researchers are still focusing on modifying planar structures to generate 3D flow characteristics, thereby alleviating the complexities caused by full-fledged 3D designs.

Many planar passive micromixers aim to generate circulating flows by means of various geometric structures. Among them, baffles are widely accepted to demonstrate efficacy in mixing enhancement. For instance, Tsai et al. [38] placed baffles radially in a curved microchannel to induce vortices in multiple directions. Sotowa et al. [39] utilized indentations and baffles to induce secondary flow in deep micro-channel reactors. Borgohain et al. [40] embedded curved ribs in a straight microchannel to promote circulating flow in the cross section, enhancing mixing performance at 0.125≤Re≤64. Chung et al. [41] implemented rectangular baffles with side gaps to induce vortices within each mixing chamber, achieving over 90% of the mixing index at both Re < 0.1 and Re > 40. Raza et al. [42] arranged baffles just after each SAR unit to induce vortices, resulting in significant mixing enhancement across a wide range of Reynolds number (0.1 ≤Re≤80). Xia et al. [43] investigated a planar micromixer with baffles and gaps, showing mixing efficiency as high as 94% at extremely low (Re = 0.1) or high Reynolds numbers (Re≥40). Ahmadi et al. [44] introduced baffles into the side walls of curved serpentine micromixers to enhance mixing performance at relatively low Reynolds numbers (1 ≤Re≤50), achieving a mixing index of 0.98 at a Reynolds number of 20. While these planar structures have shown some enhancement either in the molecular dominant or convection dominant regimes, further improvement is needed, particularly in the transition regime of Reynolds numbers (0.2 ≤Re≤20). 

As the conventional geometric structures such as baffles focused on generating circulating flows in the cross-flow direction, a novel geometric modification aimed at generating circulating flows in the transverse direction would be beneficial to obtain mixing enhancement in the transition regime. Recently, the submergence of planar structures has proved efficacy in enhancing the mixing performance, particularly in the transitional mixing regime. This design approach offers the additional benefit of reduced pressure drop. For example, Hsiao et al. [45] submerged pairs of winglets to generate longitudinal vortices, achieving noticeable improvements in mixing across a wide range of Reynolds numbers (0.125≤Re≤64). Makhsuda et al. [30] demonstrated that submerged planar baffles induced the formation of secondary vortices in the transverse direction, leading to 1.38 times higher mixing index compared to the case with no submergence at Re = 5. Additionally, submerged planar structures are easily constructed through microfabrication techniques like Xurography [46] and a single-step dual-layer photolithography [47], offering a simpler alternative to complex 3D micromixers. For example, Xurography technique conveniently fabricates a submerged structure using thin, pressure-sensitive double-sided adhesive flexible films. Martínez-López et al. [48] have demonstrated how a passive micromixer with submerged structures can be effortlessly constructed by assembling the tailored film onto planar structures. 

In this research, we have developed a novel passive micromixer aimed at enhancing mixing performance specifically within the transition regime. The present micromixer comprises six mixing units, each featuring two opposing circular microchannels with six submerged circular baffles. To optimize mixing enhancement, we designed three different arrangements of submerged circular baffles and evaluated their respective mixing performance. The evaluation of mixing performance involves computing the DOM at the outlet and the required pressure load between the inlets and outlet. All numerical simulations were carried out using ANSYS^®^ Fluent 2021 R2 [49]. 

## 2. Governing Equations and Computational Procedure

The fluid flow and mixing characteristics of present micromixer were simulated using a commercial software, ANSYS^®^ FLUENT 2021 R2 [49]. This software solves continuity, Navier–Stokes equations, and an advection–diffusion equation for mixing by the finite volume method. Therefore, we utilized the following continuity and Navier–Stokes equations:

(1) $$\left(\vec{u}\cdot \nabla \right)\vec{u}=-\frac{1}{\rho }\nabla p+\nu \nabla^{2}\vec{u}$$

(2) $$\nabla \cdot \vec{u}=0$$

where $\vec{u}$, *p*, and *ν* are the velocity vector, pressure, and kinematic viscosity, respectively. The simulation of mixing evolution involves solving an advection–diffusion equation given by

(3) $$\left(\vec{u}\cdot \nabla \right)\varphi =D\nabla^{2}\varphi$$

where *D* and *φ* are the mass diffusivity and mass fraction of a fluid A, respectively.

For discretizing the convective terms in Equations (1) and (3), we employed the QUICK (quadratic upstream interpolation for convective kinematics) scheme, well known for its third-order accuracy interpolation. Given that the present micromixer has two inlets, denoted as inlet 1 and inlet2, we set the mass fraction of fluid A to *φ* = 1 at inlet 1 and *φ* = 0 at inlet 2. This indicates that fluid A is introduced at inlet 1, while fluid B is introduced at inlet 2. The outlet was subjected to the outflow condition and a uniform velocity distribution was assumed at the two inlets. Additionally, we specified the no-slip boundary condition along the walls. 

We utilized two metrics, degree of mixing (DOM) and mixing energy cost (MEC), to assess the mixing performance of the present micromixer. DOM is defined as follows;

(4) $$DOM=1-\frac{1}{\xi }\sqrt{\sum_{i=1}^{n}\frac{{\left(\varphi_{i}-\xi \right)}^{2}}{n}},$$

where *φ_i_* represents the mass fraction of fluid A in the *i*th cell and *n* denotes the total number of cells. *ξ* = 0.5 represents the complete mixing of two fluids. MEC, employed to evaluate the effectiveness of the present micromixer, is defined as follows [50,51]:

(5) MEC=∆pρumean2DOM×100,

where $u_{mean}$ represents the average velocity at the outlet, and ∆p is the pressure load between the inlet and the outlet.

The fluid properties, including density, diffusion coefficient, and viscosity, were assumed to be equal to those of water with the following values: *ρ* = 997 kg/m^3^, *D* = 1.0 × 10^−10^ m^2^s^−1^, and *ν =* 0.97 × 10^−6^ m^2^s^−1^, respectively. The Reynolds number is defined as $Re=\frac{\rho U_{mean}d_{h}}{\mu }$, where $\rho ,\;U_{mean},\;d_{h},\;and\;\mu$ represent the density, mean velocity at the outlet, hydraulic diameter of the outlet channel, and absolute viscosity of the fluid, respectively. Additionally, the corresponding Schmidt (Sc) number, defined as the ratio of the kinetic viscosity and mass diffusivity of the fluid, is approximately 10^4^.

## 3. Validation of the Numerical Study

In simulating flows with high Schmidt (Sc) numbers, careful consideration of numerical diffusion is essential to maintain the accuracy of numerical solutions. Several numerical strategies have been explored to obtain quantitatively more rigorous numerical solutions. These approach include the use of particle-based simulation methodologies such as the Monte Carlo method [52,53], and the reduction in the cell Peclet number for grid-based methods. In grid-based approaches, the cell Peclet number, defined as $Pe_{c}=\frac{U_{cell}l_{cell}}{D}$, plays a pivotal role, with $U_{cell}$ and $l_{cell}$ representing the local velocity of flow and cell size, respectively. For example, Bayareh [54] recommended limiting the cell Peclet number $Pe_{c}\leq 2$ to obtain a numerical solution with negligible numerical diffusion effects. However, both approaches may render the simulation impractical within the scope of studies like the present one. In most numerical studies, a practical approach involving a grid independence test is preferred. This alternative entails comparing numerical solutions with corresponding experimental data [27,55], to ensure the reliability of numerical solutions. This pragmatic approach is employed in this paper.

**Figure 1:**
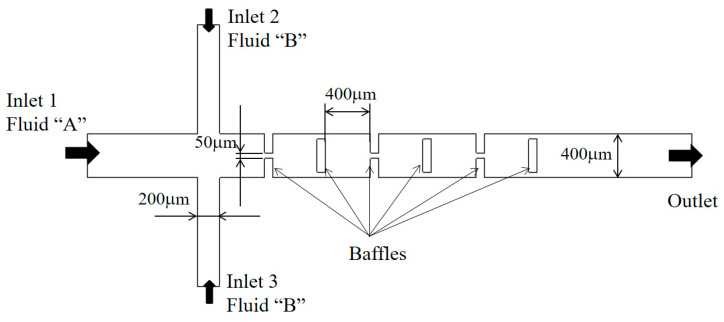
Diagram of the micromixer experimented by Chung et al. [41].

The micromixer examined by Chung et al. [41] was utilized to validate the present numerical approach. Figure 1 depicts a schematic diagram of the micromixer, consisting of three mixing units. Each mixing unit is composed of three rectangular baffles, with each baffles having a thickness of 80 μm. Since each baffle is shorter than the width of the micromixer, it creates a gap. Specifically, the first two baffles form a gap in the center, while the third baffle generates two gaps around its edges as illustrated in Figure 1. The dimensions of the inlets are as follows: the width of inlet 1 and the two side inlets, inlet 2 and inlet 3, are 400 μm and 200 μm, respectively. The depth of the micromixer is specified as 130 μm.

The fluid properties are specified as density *ρ* = 997 kg/m^3^, diffusion coefficient *D* = 3.6 × 10^−10^ m^2^s^−1^, and viscosity *ν* = 0.89 × 10^−6^ m^2^s^−1^, respectively. Consequently, the Schmidt (Sc) number is approximately 2472. The simulation was carried out and compared with the corresponding experimental data at Reynolds number Re = 60. Here, the Reynolds number is defined as $Re=\frac{\rho U_{mean}d_{h}}{\mu }$, with $\rho ,{\;U}_{mean},{\;d}_{h},and\;\mu$ representing the density, the mean velocity at the outlet, the hydraulic diameter of the outlet channel ($d_{h}=196.2\;\mu m$), and the dynamic viscosity of the fluid, respectively. Structured hexahedral cells were employed to mesh the computational domain, with a total number of cells approximately equal to 3.75 million.

**Figure 2:**
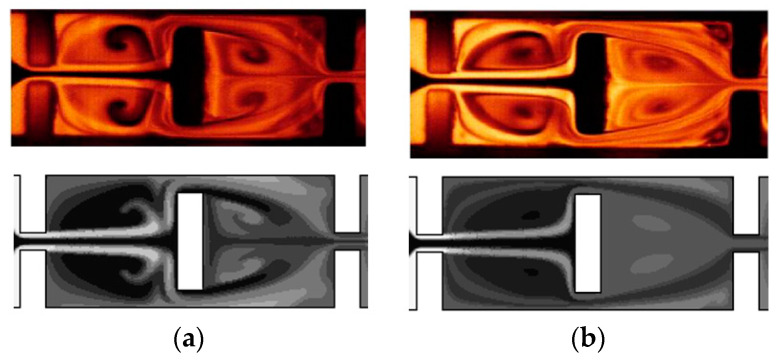

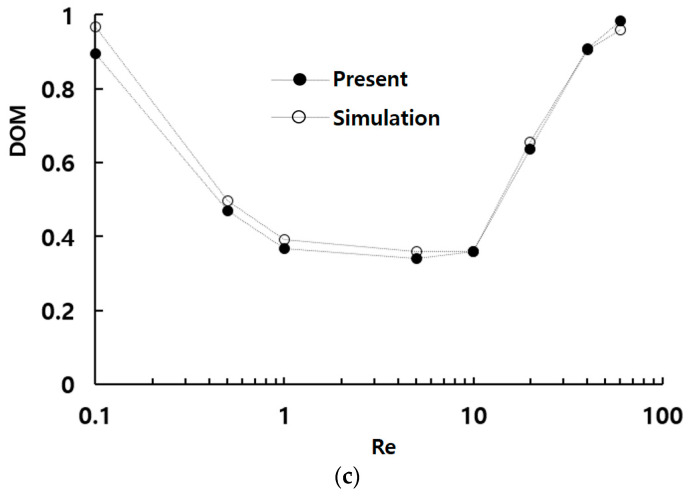
Comparison of mixing performance for Re = 60: (**a**) mixing image at z = 32.5 μm, (**b**) mixing image at z = 65 μm, and (**c**) DOM as a function of Reynolds number: simulation by Chung et al. [41].

Figure 2 compares the simulated mixing performance with the corresponding experimental and numerical results by Chung et al. [41]. The mixing images at the two different depths are depicted in Figure 2a,b, where the upper image in each figure represents the experimental data by Chung et al. [41]. The comparison reveals that the mixing process is significantly dependent on depth, showing strong mixing in the transverse direction. The present numerical simulation successfully captures crucial mixing features, including the formation of vortices around short baffles. Figure 2c provides a quantitative comparison between the present study and the simulation results by Chung et al. [41]. In our study, we employed 4.42 × 10^6^ cells, whereas Chung et al. [41] used 1.86 × 10^6^. Therefore, the present simulation employed approximately 2.4 times the number of cells compared to Chung et al. [41]. Both results demonstrate the same behavior of DOM as a function of the Reynolds number, confirming that mixing mechanism was properly simulated across the entire range of Reynolds numbers.

The discrepancies between them are primarily due to differences in the numerical scheme such as the discretization methods and the number of cells used. 

## 4. Present Micromixer with Circulation Promoters

**Figure 3:**
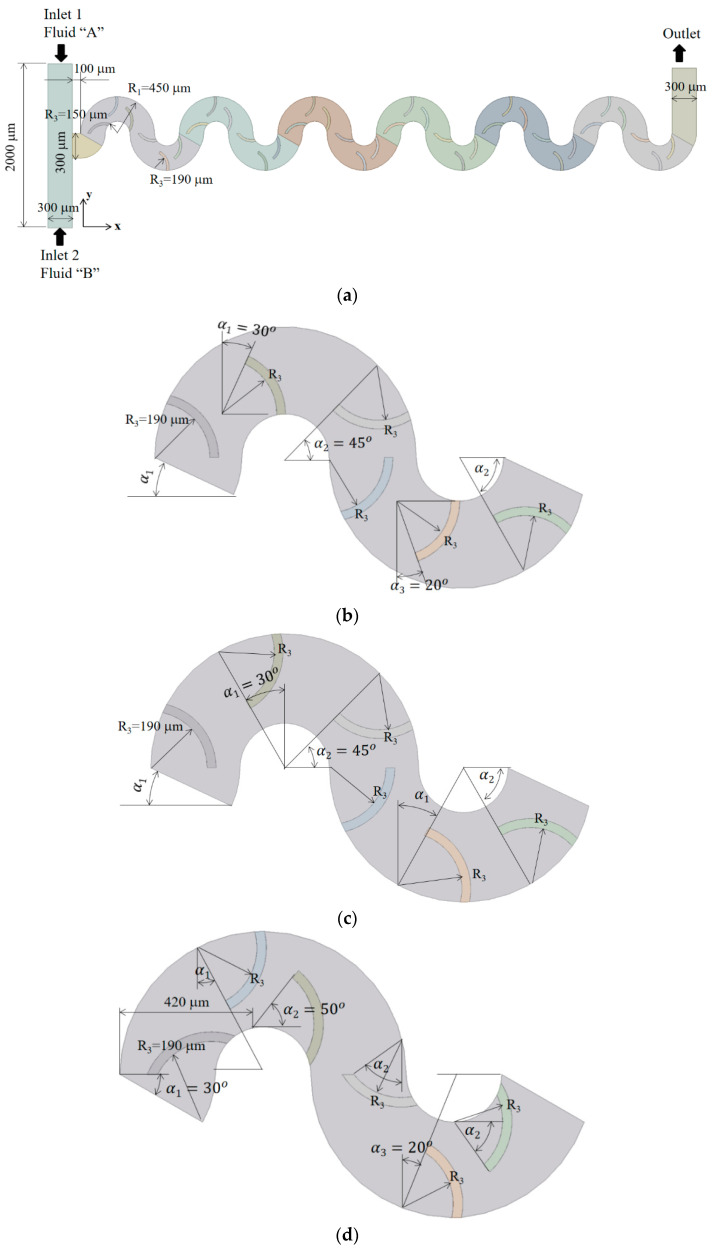
Schematic diagram of present micromixer: (**a**) front view, (**b**) Case 1, (**c**) Case 2, and (**d**) Case 3.

The present micromixer consists of six mixing units, as illustrated in Figure 3a. Each mixing unit spans 360 degrees with a radius of 300 μm and contains six circular baffles. Each circular baffle is characterized with its inner radius of 190 μm and thickness of 30 μm, arranged along either of the concave and convex circular walls. Since the height of circular baffles is 60 μm shorter than that of the microchannel, they are submerged in the z-direction. These submerged circular baffles are utilized as circulation promoters inside each mixing unit. Three different arrangement of circulation baffles were designed inside a circular microchannel. Accordingly, these mixing cells are expected to promote circulatory flows around circular baffles in different ways. The results demonstrate the effectiveness of submerged circular baffles as circulation promoters to enhance mixing performance. 

The inlet and outlet branches of the present micromixer feature a rectangular cross section, measuring 300 μm in width and 200 μm in depth. Inlet 1 and inlet 2 span 1000 μm each in length, while the outlet branch is 700 μm long. The two inlets are positioned opposite to each other so that the primary mixing process occurs in the subsequent mixing units. The total axial length of the present micromixer is approximately 7.3 mm.

Figure 3b–d depict three distinct arrangements of submerged circular baffles. Figure 3b provides an in-depth view of Case 1, featuring four baffles attached to the concave wall and two baffles attached to the convex wall. Each baffle is circular, with an inner radius of 190 μm and a thickness of 30 μm. The baffles attached to the concave wall guides the flow inward, while those attached to the convex wall pushes the flow outward. Consequently, the flow is expected to alternate between inward and outward directions it passes through the circular baffles. In contrast, Case 2 situates all baffles on the concave walls, as depicted in Figure 3c. Given that the flow along a circular microchannel naturally moves toward the concave wall due to centrifugal force, the circular baffles attached to the concave wall are expected to enhance mixing around them. For Case 3, shown in Figure 3d, more baffles are attached to the convex wall. At the entry to each mixing unit, a concave wall transitions to a convex wall due to the waviness of the microchannel. This implies that the fluid flows radially outward. As a result, the first baffle in each mixing unit impedes the flow, potentially causing noticeable flow disturbances. This flow phenomenon is the main design consideration of Case 3.

**Figure 4:**
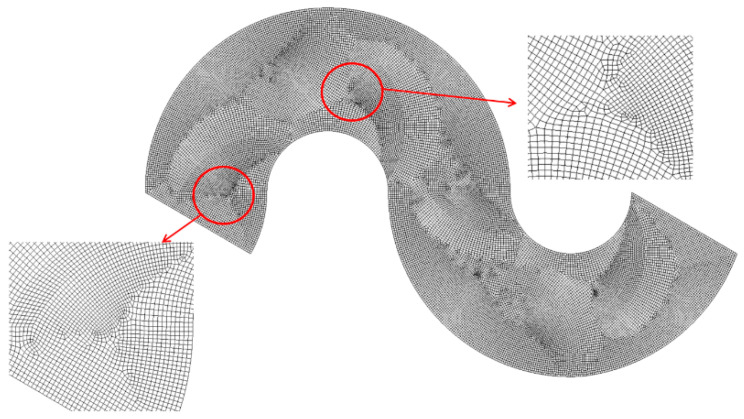
Example of mesh in a mixing unit.

The micromixers shown in Figure 3 were meshed using an appropriate number of cells. To mitigate numerical diffusion, the size of each cell was elaborately determined through a series of preliminary simulations. When generating the mesh, the edge size of each cell was constrained below a specified value, ranging from 4 μm to 6 μm. This constraint resulted in cell numbers ranging from 4 × 10^6^ to 13.7 × 10^6^. The simulation to determine mesh size was conducted at Re = 0.5. Figure 4 presents a magnified view of the mesh within a mixing unit. The choice of cell type can significantly affect the accuracy of numerical solutions, as demonstrated by Okuducu et al. [56]. Hexahedral cells are strongly recommended over prism and tetrahedral cells. In the present simulations, structured hexahedral were predominantly employed, as depicted in Figure 4. The number of prism cells was minimized, while tetrahedral cells were entirely avoided in this simulation.

The uncertainty of the numerical solution was assessed through the grid convergence index (GCI) [57,58], based on the simulation results. The GCI was computed using the following formula:

(6) $$GCI=F_{s}\frac{\left|\varepsilon \right|}{r^{p}-1},$$

where *F_s_*, *r*, and *p* represent the safety factor of the method, grid refinement ratio, and the order of accuracy of the numerical method, respectively. ε is determined by the equation:

(7) $$\varepsilon =\frac{f_{coarse}-f_{fine}}{f_{fine}},$$

where *f_coarse_* and *f_fine_* are the numerical solutions obtained with a coarse grid and fine grid, respectively. In this study, *F_s_* was set at 1.25, following the recommendation of Roache [57]. The edge size limit was adjusted to 4 μm, 5 μm, and 6 μm, resulting in corresponding cell counts of 2.14 × 10^6^, 5.2 × 10^6^, and 10.4 × 10^6^, respectively. After evaluating the GCI of the computed DOM, it was found that the GCI is approximately 0.6% when using an edge size of 5 μm. Consequently, the edge size of 5 μm was chosen to mesh the computational domain because of its favorable GCI value, ensuring a suitable balance between accuracy and computational cost.

## 5. Mixing Performance of Present Micromixers

Over a broad range of Reynolds numbers from 0.1 to 80, the mixing performance of three distinct designs was evaluated in terms of DOM and associated pressure drop. For these numerical evaluations, the uniform velocity at the two inlets was varied from 0.209 mm/s to 0.167 m/s, corresponding to volume flow rate ranging from 1.5 μL/min to 1205.6 μL/min. We computed the DOM at the outlet, while the associated pressure drop indicates the difference between the inlets and the outlet. 

**Figure 5:**
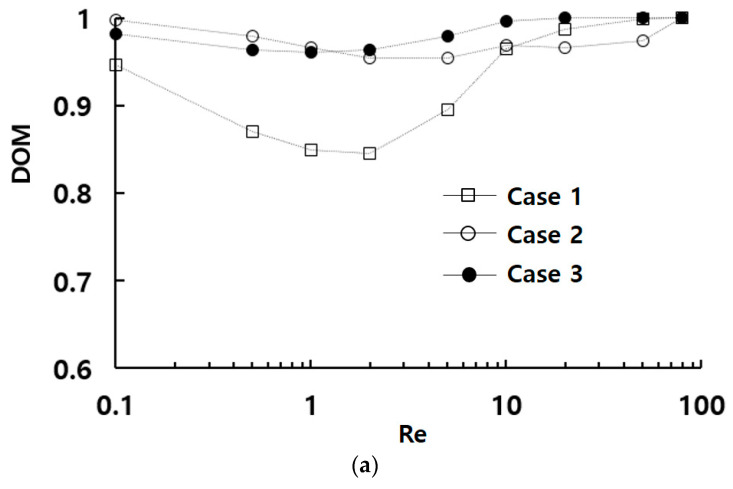

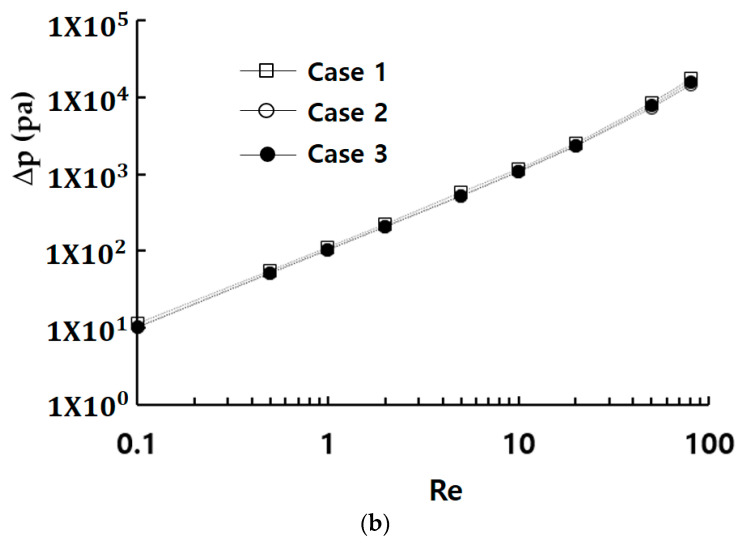
Comparison of mixing performance of three configurations: (**a**) DOM vs. Re, and (**b**) Δp vs. Re.

Figure 5 presents a comparison of the mixing performance of three designs in terms of DOM and the associated pressure drop across Reynolds numbers ranging from 0.1 to 80. Despite all three designs exhibiting DOM higher than 0.84 across the entire range of Reynolds numbers, there is a noticeable dependence on the arrangement of submerged circular baffles. Case 2 demonstrates the most favorable DOM performance for Re≤1, while Case 3 exhibits the highest DOM when the Reynolds number exceeds approximately 1. Specifically, the DOM of Case 2 is approximately 12% higher compared to Case 1 at Re = 0.5, whereas the DOM of Case 3 surpasses that of Case 1 by approximately 13% at Re = 2. However, the difference between Case 2 and Case 3 is relatively marginal. The DOMs of Case 2 and Case 3 are higher than 0.95 over the entire range of Reynolds numbers. Meanwhile, the associated pressure drop is the most advantageous for Case 2. The pressure drop in Case 2 is reduced by 8% to 16% compared to Case 1, while that in Case 3 is decreased by 8% to 9%, across Reynolds numbers ranging from 0.1 to 80. Consequently, Case 2 could be considered the optimal choice for general mixing process applications where the pressure drop is crucial, provided the DOM exceeds a certain threshold, such as 0.9. Conversely, in applications where maintain a high value of 95% complete mixing across a wide range of Reynolds number is essential [31], Case 3 might be a preferable option.

**Figure 6:**
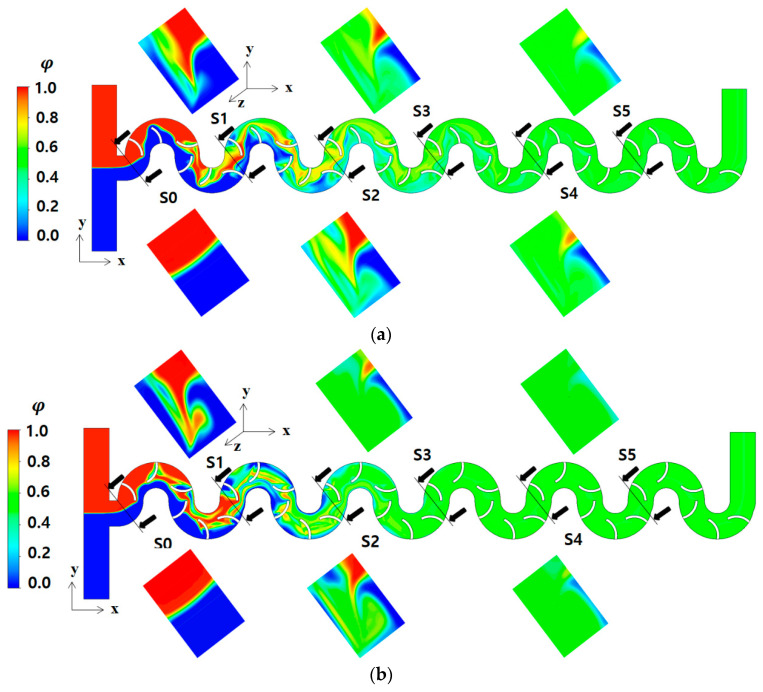

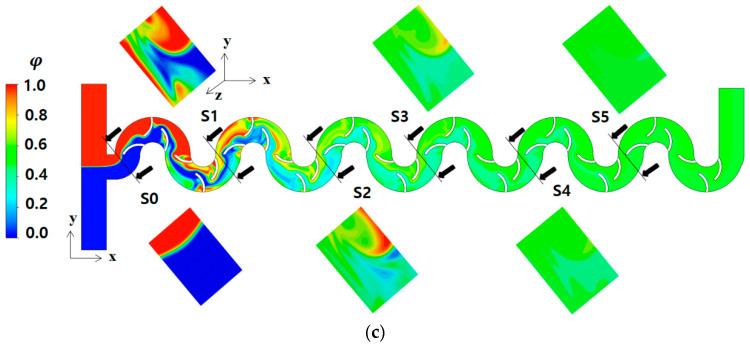
Evolution of mixing along the micromixer at Re = 2: (**a**) Case 1, (**b**) Case 2, and (**c**) Case 3. Non-proportional.

Figure 6 compares the mixing evolution within the three configurations along the micromixers at Re = 2. The concentration contours in the frontal view are obtained at z = 100 μm, while the cross sections from S0 to S5 correspond to interfaces between two consecutive mixing units. The concentration contours on section S1 show a noticeable dependence on the arrangement of circulation promoters. In Case 2, a sharp trail of interfaces in the diagonal direction suggests vigorous mixing along the interface. A similar pattern is observed on cross section S2, indicating consistent flow characteristics governs mixing. 

For Case 2, the circulation promoters are placed to guide fluid flow from a concave wall to the opposite convex wall. Case 1 shows a similar pattern of mixing evolution along the micromixer, but the interface on cross section S1 is less distinct compared to Case 2. This explains why the DOM of Case 1 is lower than that of Case 2, as seen in Figure 5. In contrast, Case 3 exhibits a different pattern of mixing evolution. The interface between the two fluids forms diagonally from the right lower corner to the left upper corner. Unlike other cases, more circulation promoters are placed on the convex wall. This configuration guides fluid flow from one concave wall to another concave wall on the opposite side at each junction of two mixing units. Since the concave wall has a larger radius than the convex wall, the flow disperses more easily in the radial direction of centrifugal force. Therefore, Case 3 performs the best in the mixing regime where the convection mixing mechanism plays a role. Conversely, Case 2 achieves the best mixing performance in the range of Reynolds numbers where the diffusion mixing mechanism is dominant.

**Figure 7:**
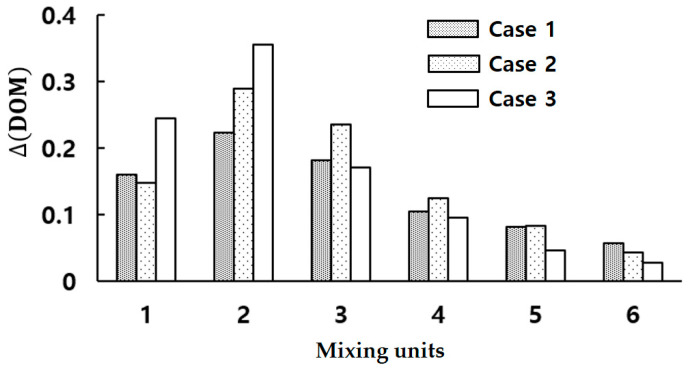
Comparison of DOM increment in each mixing unit for three configurations at Re = 2.

**Figure 8:**
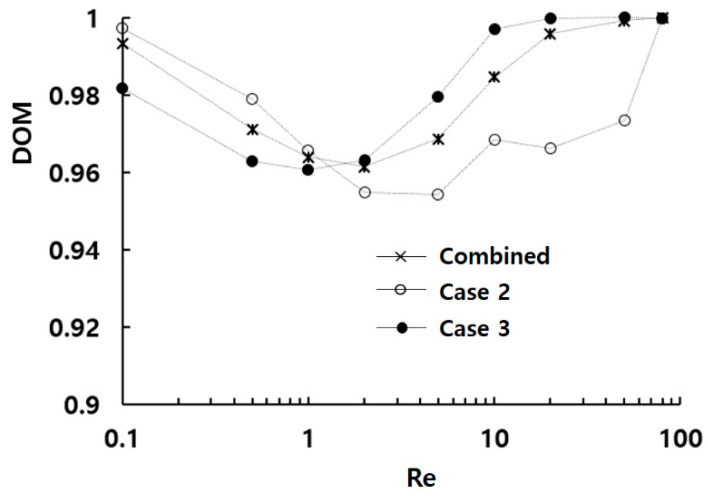
Comparison of the DOM performance of a combined micromixer with Case 2 and Case 3.

Figure 7 compares the DOM increment of the three configurations in each mixing unit at Re = 2. It reveals that active mixing occurs primarily in the first two mixing units, with a rapid decrease afterward. Another noticeable finding is that Case 2 outperforms Case 3 starting from the third mixing unit. In addition, Case 2 design performs better in the range of Reynolds numbers, Re≤1, while Case 3 is favorable for Re>1. A combined configuration of Case 2 and Case 3 could result in a more balanced DOM performance across the entire range of Reynolds numbers. Figure 8 presents the mixing performance of a micromixer that combines Case 3 design in the first three mixing units and Case 2 design in the remaining mixing units. This combined design leverages the outperformance of both configurations to enhance the mixing performance across a broad range of Reynolds numbers.

**Figure 9:**
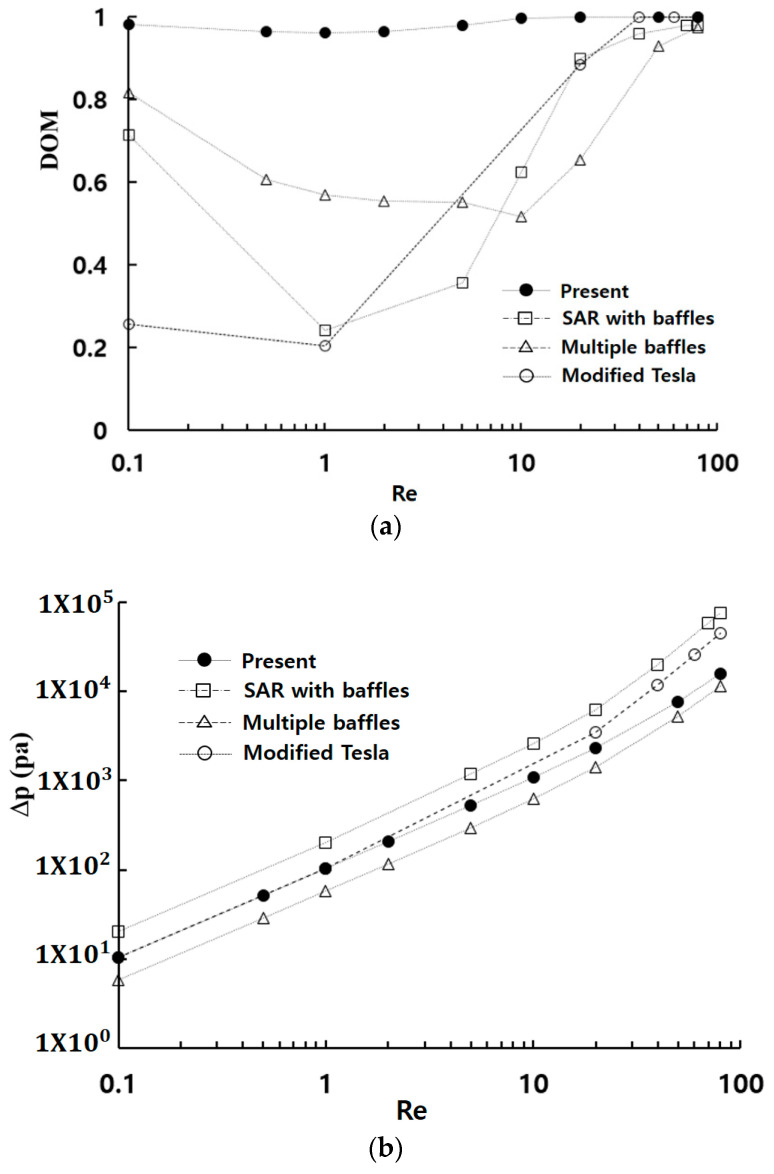
Comparison of the mixing performance of Case 3 with several passive micromixers: (**a**) DOM vs. Re, and (**b**) Δp vs. Re.

Figure 9 provides a comparative evaluation of the mixing performance of Case 3 against several passive micromixers: a SAR micromixer with baffles [42], a micromixer with multiple baffles [30], a modified Tesla micromixer [59]. All four passive micromixers were simulated under similar boundary conditions and physical properties. The submergence scheme for multiple baffles shows has a noticeable impact on DOM, with the result depicted in the figure showing the best performance [30]. The present micromixer of Case 3 shows a significant enhancement of DOM in the entire range of Reynolds numbers (0.1 ≤Re≤80), maintaining a DOM larger than 0.96 throughout the entire Reynolds number range. 

At Re = 1, the DOM of Case 3 is 3.7 times larger than that of the modified Tesla micromixer. This kind of enhancement is especially noticeable in the low and intermediate range of Reynolds numbers where the molecular diffusion plays a role in mixing process. For example, at Re = 1, the DOM of Case 3 is approximately 0.96, whereas the modified.

Tesla micromixer [59], a SAR micromixer with baffles [42], and a micromixer with multiple baffles [30] achieve DOM values of 0.2, 0.24, and 0.86, respectively. This result indicates that circulation promoters are a promising design component for enhancing mixing performance in the molecular diffusion and transition regime. Generally, passive micromixers achieve mixing enhancement at the expense of an increased pressure drop. Therefore, pressure drop should also be considered in evaluating mixing performance in terms of DOM. In Figure 9b, the SAR with baffles by Raza et al. [42] shows the highest pressure drop among the four passive micromixers, while the multiple baffles by Makhsuda et al. [30] requires the least pressure drop. The pressure drop in the present micromixer falls between them. This result, along with the highest value of DOM, implies that the submerged circulation promoters proposed in this paper are the most cost-effective for achieving mixing enhancement, especially in the low and intermediate range of Reynolds numbers (Re<40).

## 6. Conclusions

In this paper, a passive micromixer equipped with circulation promoters was proposed and its mixing performance was analyzed in terms of DOM and the associated pressure drop. The present micromixer comprises six sinusoidal mixing units, each containing six circulation promoters. These promoters are circular baffles placed along the micromixer walls, making their arrangement the principal design parameter. To assess the effectiveness of circulation promoters, we proposed three different configurations and simulated them over a broad range of Reynolds numbers using ANSYS^®^ Fluent 2021 R2.

The proposed micromixer shows a significant enhancement of DOM within the low and intermediate range of Reynolds numbers (Re<40) compared to other passive micromixers such as the modified Tesla micromixer, a passive micromixer with multiple baffles, and a SAR micromixer with baffles. The DOM of the present micromixer exceeds 0.96 across the entire range of Reynolds numbers (Re≤80). Specifically, the DOM of Case 3 is 3.7 times greater than that of the modified Tesla micromixer at Re = 1. Moreover, the required pressure drop is comparable with those of other passive micromixers. These results demonstrate that the circulation promoters are a potential element for achieving high mixing performance throughout a wide range of Reynolds numbers.

In the low range of Reynolds numbers (Re≤1), the mixing enhancement is primarily due to circulation promoters directing fluid flow from a concave wall to the opposite convex wall. Conversely, in the intermediate range of Reynolds numbers (2≤Re<40), the mixing enhancement results from fluid flowing from one concave wall to another concave wall on the opposite side. This flow characteristics is materialized by placing more circulation promoters on the convex wall. Since the concave wall has a larger radius than that of convex wall, the flow can disperse easily, as Reynolds number increases, in the radial direction of centrifugal force. These flow characteristics underscore the superior effectiveness of circulation promoters over conventional solid geometries like circular cylinders, in enhancing the mixing performance.

The circulation promoters have demonstrated their effectiveness in enhancing the mixing performance of passive micromixers, over a broad range of Reynolds numbers (0.1 ≤Re<80). Compared to other typical passive micromixers, the circulation promoters result in a notable enhancement of DOM, particularly within the low and intermediate Reynolds number range of Re<40. Furthermore, the DOM of the present micromixer based on circulation promoters remains consistently high, surpassing 0.96 across the entire Reynolds number range (0.1≤Re≤80), with a pressure drop comparable to other planar micromixers. 

## Data Availability

The original contributions presented in the study are included in the article, further inquiries can be directed to the corresponding author.
